# Real-World Patient Experience of Pexidartinib for Tenosynovial Giant-Cell Tumor

**DOI:** 10.1093/oncolo/oyad282

**Published:** 2023-10-24

**Authors:** Feng Lin, Winghan Jacqueline Kwong, Irene Pan, Xin Ye, Dong Dai, William Tap

**Affiliations:** Health Economics and Outcomes Research, Daiichi Sankyo, Inc., Basking Ridge, NJ, USA; Health Economics and Outcomes Research, Daiichi Sankyo, Inc., Basking Ridge, NJ, USA; Epidemiology and Real-World Evidence, United BioSource LLC, Blue Bell, PA, USA; Health Economics and Outcomes Research, Daiichi Sankyo, Inc., Basking Ridge, NJ, USA; Health Economics and Outcomes Research, Daiichi Sankyo, Inc., Basking Ridge, NJ, USA; Sarcoma Medical Oncology Service, Memorial Sloan Kettering Cancer Center, New York, NY, USA

**Keywords:** pexidartinib, tenosynovial giant-cell tumor, patient-reported outcome, symptom change

## Abstract

**Background:**

Pexidartinib (Turalio) is the only systemic therapy approved by the FDA for the treatment of adult patients with symptomatic tenosynovial giant-cell tumor (TGCT) associated with severe morbidity or functional limitations, and not amenable to improvement with surgery. This study assessed patient-reported treatment experiences and symptom improvement among patients receiving pexidartinib.

**Methods:**

A cross-sectional, web-based survey collected data on demographics, disease history, pexidartinib dosing, and symptoms before and after pexidartinib use.

**Results:**

Of 288 patients enrolled in the Turalio REMS program in May 2021, 83 completed the survey: mean age was 44.2 years, 62.7% were female, and most common tumor sites were in knee (61%) and ankle (12%). Mean initial dose was 622 mg/day: 29 patients reported reduction from initial dose and 8 had dose reduction after titrating up to a higher dose. At the time of survey completion, median time on pexidartinib was 6.0 months; 22 (26.5%) patients discontinued pexidartinib due to physician suggestion, abnormal laboratory results, side effect, or symptom improvement. Compared with before pexidartinib initiation, most patients reported improvement in overall TGCT symptom (78.3%) and physical function (77.2%) during pexidartinib treatment. Significant improvement was reported during pexidartinib treatment in worst stiffness numeric rating scale (NRS) (3.0 vs. 6.2, *P* < .05) and worst pain NRS (2.7 vs. 5.7, *P* < .05).

**Conclusion:**

Findings from this cross-sectional survey confirmed the benefit of pexidartinib in improving symptoms and functional outcomes among patients with symptomatic TGCTs from the patients’ perspective. Future research is warranted to examine the long-term benefit and risk of pexidartinib.

## Teaser

This study assessed patient-reported treatment experiences and symptom improvement among patients receiving pexidartinib, the only systemic therapy approved by the FDA for treatment of this condition.

**Implications for Practice**

Pexidartinib is available to US patients who are registered in the Turalio Risk Evaluation and Mitigation Strategy (REMS) program. In this web-based, cross-sectional survey of adult patients in the Turalio REMS program, we compared data on demographics, medical history, and patient-reported outcomes to the phase III ENLIVEN clinical trial population. Our analysis found that the clinical trial population represented real-world patients. However, the longer time from diagnosis to pexidartinib initiation, higher rates of surgery, and off-label imatinib use in the real-world setting suggest that pexidartinib fulfills an unmet medical need of effective systemic treatment.

## Introduction

Tenosynovial giant-cell tumor (TGCT) is a rare, locally aggressive, typically benign neoplasm arising from the synovium, bursae, or tendon sheath.^1,2^ TGCTs can be classified into either localized or diffuse types: localized tumors are generally indolent and affect smaller joints, whereas diffuse disease presents as multiple nodules throughout the synovium with poorly defined borders, mostly affecting large joints.^3^ And for severity, TGCT can be further classified into 4 subtypes (mild localized, severe localized, moderate diffuse, and severe diffuse) based on diffuse or localized TGCT, intra- or extra-articular involvement, and involvement of muscles, tendons, and ligaments.^4^ The incidence of TGCT worldwide is estimated to be 11 to 50 per million, with localized type being more prevalent.^5-7^ Although TGCT affects individuals of all ages, it is most commonly documented in working-age adults and can be associated with severe morbidity.^5,8^ Typical symptoms include pain, stiffness, swelling, and limited range of motion; however, disease presentation varies across patients.^8,9^

Although not life-threatening, advanced TGCT could have a debilitating impact on quality of life.^9^ Surgery is the current standard of care for most TGCT patients^10^; however, complete excision might be challenging, due to the lack of clearly defined boundaries or challenging location for removal, or the presence of intra-articular extensions.^9,11^ Disease recurrence was reported in up to 15% of patients with localized TGCT and up to ~50% of patients with diffuse disease.^11-13^ Residual and persistent disease might result in bone erosion and long-term joint dysfunction. Moreover, recurrent disease often requires repeated surgeries, leading to increased morbidity and impaired quality of life.^9^

Pexidartinib (Turalio, Daiichi Sankyo, Inc.), an oral small-molecule tyrosine kinase inhibitor with strong selective activity against colony-stimulating factor 1 receptor (CSF1R), has become the only systemic therapy approved by the Food and Drug Administration since August 2, 2019, for the treatment of adult patients with symptomatic TGCTs associated with severe morbidity or functional limitations that were not amenable to improvement with surgery.^14^ The approval was based on the double-blind, randomized, placebo-controlled, phase III ENLIVEN trial, which demonstrated a statistically significant improvement of 38% (95% CI, 27%-50%) in overall response rate (ORR) and meaningful improvements in physical function and stiffness at week 25 in patients randomized to pexidartinib compared with placebo.^15,16^ The most common side effects in the pexidartinib group included hair color changes, fatigue, and nausea; serious adverse reactions included abnormal liver tests, including increase in aspartate aminotransferase, ala9 aminotransferase, cholestatic hepatotoxicity, or liver failure requiring liver transplant or possibly causing death.^15^ Because of the identified risk of rare, mixed or cholestatic hepatotoxicity, pexidartinib is available only through the Turalio Risk Evaluation and Mitigation Strategy (REMS) Program in the US.^14^

With prolonged follow-up of a median of 31.2 (range: 2-66) months of ENLIVEN patients, pexidartinib maintained its clinical benefit, with an increase in ORR to 61% (95% CI, 48%-72%), and no new safety signals were observed after long-term treatment.^17^ Findings from the long-term data-cut also demonstrated sustained improvement in patient-reported physical function and stiffness after 50 weeks of pexidartinib treatment.^16,18^

With demonstrated efficacy in ENLIVEN, it remains important to understand how pexidartinib is prescribed, whether it is effective in disease control, and how well patients tolerate the treatment in real-world clinical practice. Currently, there are few data on the effectiveness of pexidartinib from the patient’s perspective in the real-world setting. This study was designed to explore patient-reported experiences with pexidartinib as treatment for TGCT, including pexidartinib dosing, symptoms at affected joints, patient impressions of symptom change during treatment, and supportive care used before and after initiation of pexidartinib in real-world setting.

## Methods

A web-based, cross-sectional survey was administered from May to July 2021 to patients enrolled in the Turalio REMS program who had current or past experience of pexidartinib.

### Study Population

Study participants were recruited from the Turalio REMS program, and they received honorarium for completing the survey. Patients, who were at least 18 years of age, had taken at least one dose of pexidartinib, had not participated in any clinical trials for pexidartinib, and could complete questionnaires in English were eligible. If they met the eligibility criteria, participants were presented with an online informed consent form, and only those who consented proceeded to the online survey. The study protocol and questionnaire were approved by Advarra Institutional Review Board (IRB) (Columbia, MD) on March 24, 2021.

### Survey Development

A cross-sectional, web-based survey was programmed and hosted using QuestionPro (Seattle, WA), a Health Insurance Portability and Accountability Act (HIPAA)-compliant, online survey tool.

After completing the screening questions, participants were asked to provide medical history about their disease condition, including year of initial TGCT diagnosis, location of the tumors, surgical history in the affected joint, and pharmacologicalal (opioids, nonsteroidal anti-inflammatory drugs [NSAIDs], corticosteroids, and antibiotics) and nonpharmacologicalal therapies (occupational therapy, rehabilitation, physical therapy, acupuncture, and dietitian/nutritionist) used to manage their TGCT prior to starting treatment with pexidartinib.

Participants were asked to provide the date and the number of capsules taken on the first and most recent days of taking pexidartinib, and the highest number of capsules taken since treatment initiation. Participants who had stopped taking pexidartinib at the time of the survey were asked to report the number of capsules on the last day of taking pexidartinib, reasons for discontinuation, side effects experienced, and subsequent treatment planned after discontinuation of pexidartinib.

Participants were asked to select the symptoms that they experienced in the past week, followed by the 7-point Patient Global Impression of Change (PGIC) scale.^19^

Physical function was measured by a 13-item questionnaire customized to assess lower-limb function among patients with lower-extremity tumors and an 11-item questionnaire customized to assess upper-limb function among patients with upper-extremity tumors from the 121-item Patient-Reported Outcomes Measurement Information System (PROMIS), Physical Function (PF).^8,20-23^ The content validity of PROMIS-PF has been demonstrated for patients with TGCT.^20^ PROMIS-PF scores are expressed as T-scores where a higher score represents better physical function, and a score of 50 represents the average level of physical functioning in the US general population with a standard deviation of 10. Patients were then asked to rate their impression of change in physical functioning since initiating pexidartinib.

Stiffness and pain were also evaluated using the 1-item Worst Stiffness Numerical Rating Scale (NRS) and Worst Pain NRS during 3 time windows: the previous 24 hours, prior to starting pexidartinib, and while taking pexidartinib. The Worst Stiffness NRS assesses the worst stiffness ranging from 0 (no stiffness) to 10 (stiffness as bad as you can imagine), and the Worst Pain NRS assesses the worst pain ranging from 0 (no pain) to 10 (pain as bad as you can imagine).

In addition to the data collected directly from patients through the web-based survey, clinical data reported by physicians at the time of registering patients in REMS were obtained from the REMS database to describe patients’ baseline clinical characteristics.

### Data Analysis

Descriptive analysis was conducted to summarize the survey responses. Continuous data are presented as mean and standard deviation (SD), median and interquartile range (IQR); categorical variables are presented as frequency counts with percentages. Statistical analyses were performed using SAS Version 9.1.3 (SPSS, IBM Corp, Armonk, NY, US). Paired *t*-tests were performed for the Worst Stiffness and Worst Pain NRS before and during pexidartinib treatment.

## Results

### Patient Characteristics

**Figure 1.**
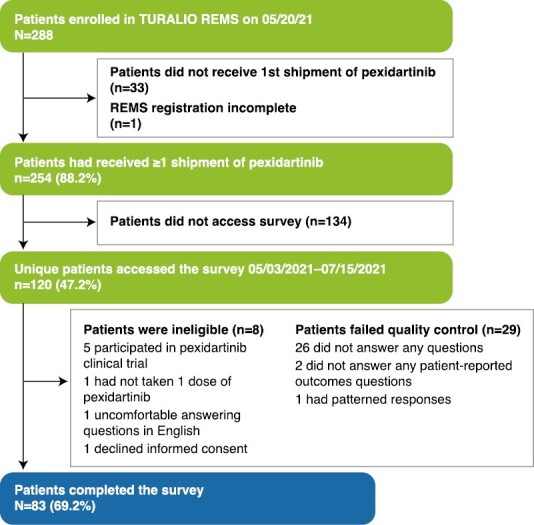
Patient attrition. REMS, Risk Evaluation and Mitigation Strategy.

The survey was fielded from May 20 to July 15, 2021. Among the 288 patients enrolled the Turalio REMS program in May 2021, 254 patients who had completed REMS registration and had a record of pexidartinib shipment date were invited: 120 unique patients had accessed the survey, and 112 were screened as eligible (Fig. 1). Twenty-nine patients were excluded from the analysis: 26 did not answer any core questions, 2 did not answer any patient-reported outcomes (PRO) questions, and 1 had patterned responses. A total of 83 patients who passed the quality assurance steps were included in the full analysis set.

Patient demographic characteristics are summarized in Table 1. The median (IQR) age at REMS enrollment was 45.0 (34.5, 55.5) years, and the cohort was predominantly female (62.7%) and White (65.1%). Geographic distribution was similar across 4 regions in the US. About 2-thirds of patients had commercial insurance coverage, and 24.0% had government insurance. The median time from TGCT diagnosis to initiation of pexidartinib was 4.0 years. Most patients had tumors in the lower extremities (88.0%), with the knee (61.4%) and ankle (12.0%) being the joints most frequently affected (Table 2). Prior to treatment with pexidartinib, 70.4% of patients underwent joint surgery (open synovectomy 57.9%, arthroscopy 36.8%, combined/2-stage synovectomy 33.3% and total joint replacement 5.3%) for TGCTs, and 25.9% received imatinib (Table 2). The majority (88.0%) of patients did not have a history of liver disease; 4 patients had diabetes, and 3 had viral hepatitis.

**Table 1.** Demographic characteristics.

Variable	Survey responders *N* = 83
Age^a^, mean (SD), y	44.2 (14.1)
Sex, *n* (%)	
Female	52 (62.7)
Male	30 (36.1)
Missing	1 (1.2)
Race, *n* (%)	
White	54 (65.1)
Black or African American	7 (8.4)
Asian	6 (7.2)
Native American	0 (0.0)
Native Hawaiian or other Pacific Islander	0 (0.0)
Other	15 (18.1)
Missing	1 (1.2)
Geographic location, *n* (%)	
Northeast	18 (21.7)
Midwest	22 (26.5)
South	20 (24.1)
West	21 (25.3)
Other	1 (1.2)
Missing	1 (1.2)
Insurance type, *n* (%)	
Medicare	5 (6.0)
Medicaid	9 (10.8)
DOD or VA	6 (7.2)
Private insurance/commercial insurance (provided by employer)	58 (69.9)
Self-ensured/Obamacare	5 (6.0)
Highest level of education completed, *n* (%)	
Less than high school	0 (0.0)
High school graduate or equivalent	5 (6.0)
Some college/university (less than 4 years)	27 (32.5)
College/university (4-year bachelor’s degree)	26 (31.3)
Postgraduate degree (eg, master’s, doctorate)	22 (26.5)
Missing	3 (3.6)
Employment status, *n* (%)	
Used, full-time	49 (59.0)
Used, part-time	6 (7.2)
Unused	7 (8.4)
Student	4 (4.8)
Homemaker	6 (7.2)
Sick leave/disabled	4 (4.8)
Other	4 (4.8)
Missing	3 (3.6)

^a^Age was extracted from the Turalio REMS program patient enrolment forms.

Abbreviations: DOD: Department of Defense; REMS: Risk Evaluation and Mitigation Strategy; SD: standard deviation; VA: Veterans Affairs.

**Table 2.** Tumor location and history of surgical intervention and systemic therapy prior to initiating pexidartinib.

Variable	Survey responders *N* = 83
Tumor location (lower extremities), *n* (%)	
Knee	51 (61.4)
Ankle	10 (12.0)
Hip	10 (12.0)
Foot	7 (8.4)
Tumor location (upper extremities), *n* (%)	
Spine	0 (0.0)
Shoulder	2 (2.4)
Elbow	2 (2.4)
Wrist	3 (3.6)
Hand/fingers	3 (3.6)
Number of prior surgeries for TGCT, *n* (%)^a^	
0	24 (29.6)
1	43 (53.1)
2	9 (11.1)
3+	5 (6.2)
Prior surgery types, *n* (%)^b^	
Open synovectomy	33 (57.9)
Arthroscopy	21 (36.8)
Combined/2-stage synovectomy	19 (33.3)
Total joint replacement	3 (5.3)
Amputation	0 (0.0)
Previous systemic therapy, *n* (%)	
None	62 (74.1)
Imatinib	21 (25.9)
Nilotinib	1 (1.2)
Hepatic medical history, *n* (%)	
No hepatic medical history	73 (88.0)
Hepatitis viral status	3 (3.6)
Family history of liver disease	2 (2.4)
Biliary tract disorder	1 (1.2)
Hypertriglyceridemia	1 (1.2)
Gall bladder disease/ gallstones/bile duct occlusion	2 (2.4)
Diabetes	4 (4.8)
Time to initiating pexidartinib from initial TGCT diagnosis	
*N*	80
*N* missing	3
Mean (SD), year	5.4 (4.64)
Median (minutes, max), year	4.0 (1, 22)

^a^% was calculated for 81 patients who reported prior surgeries for TGCT (2 not reported).

^b^% was calculated for 57 patients who had prior surgeries for TGCT, and one patient could have more than one surgeries.

Abbreviations: TGCT, tenosynovial giant-cell tumor.

### Pexidartinib Utilization Pattern

Patient-reported mean (SD) pexidartinib dose on the first day was 622 (200) mg, with 55.4% starting at the full recommended dose of 800 mg, 4.8% at 600 mg, and 34.9% at 400 mg (Table 3). Dose reduction from index dose was reported by 29 (34.9%) patients: 26 patients started at daily dose of 800 mg and 3 started at 400 mg. Dose increase was reported by 20 (24.1%) patients: 12 (14.5%) patients titrated up and remained at their highest dose, while 8 (9.6%) patients had dose reduction after titrating to their highest dose.

**Table 3.** Dosing pattern and reasons for discontinuation of pexidartinib.

Dosing pattern, *n* (%)	Survey responders (*N* = 83)
*Starting daily dose*	
800 mg	46 (55.4)
600 mg	4 (4.8)
400 mg	29 (34.9)
200 mg	4 (4.8)
*Maximum dose*	
800 mg	61 (73.5)
600 mg	8 (9.6)
400 mg	13 (15.7)
200 mg	1 (1.2)
*Dose on the day of survey completion*	
800 mg	25 (30.1)
600 mg	13 (15.7)
400 mg	21 (25.3)
200 mg	2 (2.4)
*Treatment withheld*	12 (14.4)
Not applicable: patients who had discontinued pexidartinib at the time of survey	22 (26.5)
*Dose on the last day of taking pexidartinib*	
800 mg	3 (3.6)
600 mg	2 (2.4)
400 mg	13 (15.7)
200 mg	4 (4.8)
*Dose modification*	
No change	34 (41.0)
Dose reduction	29 (34.9)
Dose titration	20 (24.1)
Titrated up and remained at highest dose	12 (14.5)
Titrated up followed by dose reduction	8 (9.6)
*Reason for discontinuation or withholding pexidartinib*	
My doctor suggested that I stop	10 (12.0)
I had abnormal lab test results	7 (8.4)
I experienced a side effect(s)	7 (8.4)
I don’t need it because my symptoms have improved	5 (6.0)
I am taking a break from pexidartinib but plan to restart	2 (2.4)
I did not like taking a medication every day	1 (1.2)
Other	8 (9.6)
*Side effect leading to discontinuation or treatment withheld*	
Hair color changes	7 (8.4)
Changes in blood liver tests	5 (6.0)
Tiredness	5 (6.0)
Swelling in and around your eyes	3 (3.6)
Increased cholesterol level in the blood	1 (1.2)
Decreased White blood cells and red blood cells	1 (1.2)
Rash	1 (1.2)
Loss of taste or changes in the way things taste	1 (1.2)
Other	4 (4.8)
*Subsequent treatment plan*	
None	13 (15.7)
Scheduled joint surgery	7 (8.4)
Imatinib	2 (2.4)

At the time of survey completion (May to July 2021), the median (IQR) time on pexidartinib was 5.95 (0.05, 11.85) months: 24 patients had been on treatment for over 12 months, 16 for between 7 and 12 months, 16 for between 3 and 6 months, and 25 for less than 3 months. Twenty-two (26.5%) patients had stopped taking pexidartinib by the day of survey: 10 patients reported complete discontinuation, and 12 had pexidartinib on hold. The mean dose on the date of survey for the 61 patients who remained on pexidartinib was 600 mg/day, with 25, 13, and 21 patients reporting 800, 600, and 400 mg/day, respectively. The mean dose was 436.4 mg on the last date of taking pexidartinib for the 22 patients who had stopped taking pexidartinib, with 3, 2, 13, and 4 patients on 800, 600, 400, and 200 mg, respectively. The most common reasons for treatment discontinuation included physician suggestion (*n* = 10), abnormal laboratory results (*n* = 7), and side effect (*n* = 7); 5 patients reported no longer needing pexidartinib because of symptom improvement (Table 3). Hair color changes, abnormal liver enzyme tests, and fatigue were the most common adverse events reported by patients who discontinued pexidartinib. Eight patients had joint surgery scheduled, and 2 patients started taking imatinib after discontinuing pexidartinib.

### Patient-Reported Outcomes

In the week before survey day, patient-reported TGCT-related symptoms included pain, stiffness, joint sound during movement, limited range of motion, warmth of the skin over the joint and swelling. The majority (78.3%) of patients reported improvement in overall joint symptoms: 27.7% reported “Very much improved,” 30.1% reported “Much improved,” and 20.5% reported “Minimally improved.” Twelve (14.5%) patients reported “No change,” and 5 (6.3%) reported worsening in overall symptoms (Table 4).

**Table 4.** Patient-reported symptoms, physical function, and overall impression of change

Variable	Survey responders (*N* = 83)
Symptoms relating to joint tumor in the past week, n (%)	
Pain or tenderness	52 (62.7)
Stiffness	43 (51.8)
Locking/popping/catching sound during movement	34 (41.0)
Limited range of motion	32 (38.6)
Warmth of the skin over the joint	24 (28.9)
Swelling	15 (18.1)
PGIC on overall symptoms since initiating pexidartinib, n (%)	
Very much improved	23 (27.7)
Much improved	25 (30.1)
Minimally improved	17 (20.5)
No change	12 (14.5)
Minimally worse	2 (2.4)
Much worse	1 (1.2)
Very much worse	2 (2.4)
Missing	1 (1.2)
PROMIS-PF aggregate (T-score) on the date of survey: n; mean (SD)	82; 44.49 (8.38)
PROMIS-PF upper extremity (T-score) on the date of survey: n; mean (SD)	10; 49.06 (8.28)
PROMIS-PF lower extremity (T-score) on the date of survey: n; mean (SD)	72; 43.86 (8.20)
PGIC on physical function since initiating pexidartinib, n (%)	
Very much improved	25 (30.1)
Much improved	24 (28.9)
Minimally improved	16 (19.3)
No change	14 (16.9)
Minimally worse	1 (1.2)
Much worse	1 (1.2)
Very much worse	2 (2.4)
Missing	1 (1.2)

Abbreviations: PGIC: Patient Global Impression of Change; PROMIS-PF: Patient-Reported Outcomes Measurement Information System-Physical Function.

The mean (SD) PROMIS-PF T-score when patients were taking pexidartinib was 44.5 (8.38) for 82 patients who responded: 43.86 (8.20) for 72 patients with tumors located in the lower extremities and 49.06 (8.28) for 10 patients with tumors located in the upper extremities. The majority (77.1%) of patients reported improvement in physical function: 30.1% reported “Very much improved,” 28.9% reported “Much improved,” and 19.3% reported “Minimally improved.”

Results on Worst Stiffness NRS and Worst Pain NRS are summarized in Fig. 3 where the scores before taking pexidartinib were recalled by participants. The mean (SD) Worst Stiffness NRS during treatment with pexidartinib was 3.0 (2.42), with a 3.2-point reduction (*P* < .001) from mean (SD) of 6.2 (2.77) before taking pexidartinib, and 73.2% patients reported Worst Stiffness NRS reduction that exceeded the clinically meaningful threshold of ≥1 point change.^24^ The mean (SD) Worst Pain NRS while taking pexidartinib was 2.7 (2.42), with a 3.0-point reduction (*P* < .001) from mean (SD) of 5.7 (2.70) before taking pexidartinib, and 65.9% patients reported Worst Pain NRS reduction that exceeded the clinically meaningful threshold of ≥2 points change.^25^

**Figure 2.**
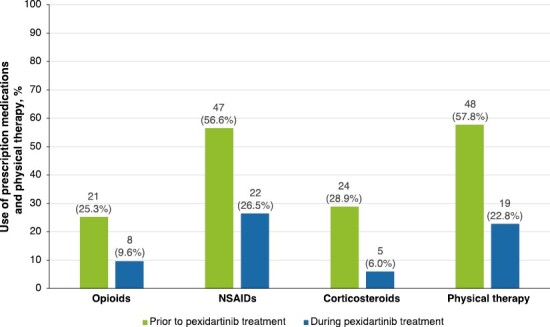
Utilization of prescription medications and physical therapy prior to and during pexidartinib treatment. NSAIDs, nonsteroidal anti-inflammatory drugs.

**Figure 3.**
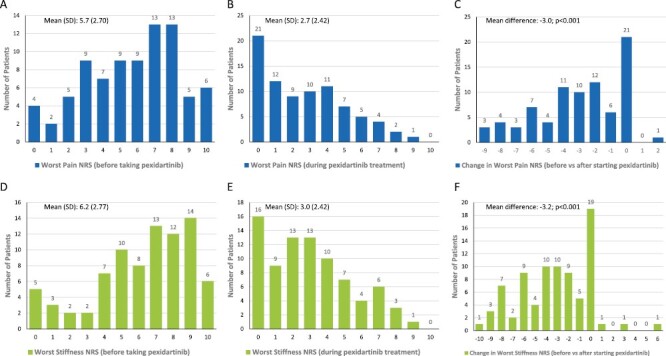
Histograms of the Worst Pain (**A**-**C**) and Worst Stiffness (**D**-**F**) NRS scores reported before and during pexidartinib treatment. Abbreviation: NRS: numeric rating scale.

### Prescription Medication and Supportive Care

NSAIDs were the most commonly prescribed medication used for symptom management before initiating pexidartinib (*n* = 47; 56.6%), followed by corticosteroids (*n* = 24; 28.9%), and opioids (*n* = 21; 25.3%) (Fig. 2). When patients were receiving pexidartinib, utilization of NSAIDs, corticosteroids, and opioids was reduced to 22 (26.5%), 5 (6.0%), and 8 (9.6%) patients, respectively. Physical therapy was also the common alternative therapy reported before initiating pexidartinib (*n* = 48; 57.8%), and fewer patients reported utilization of physical therapy (*n* = 19; 22.8%) during pexidartinib treatment.

## Discussion

To our knowledge, this survey study is the first to report real-world treatment experience with pexidartinib from a patient’s perspective. This study confirmed that TGCT has its onset in a relatively young and working patient population with a female predominance.^15,18,26,27^ The TGCT Observational Platform Project (TOPP) registry study was conducted by Bernthal et al to explore the management of TGCT in tertiary sarcoma centers.^27^ This first multinational, multicenter, prospective, observational disease registry study involved hospitals and tertiary sarcoma centers in Europe and the US.^27^ The demographic and clinical characteristics at the time of entry to the registry were reported for all patients, and patients were followed for minimum of 2 years.^27^ Similar to patients enrolled in ENLIVEN or TOPP,^15,27^ most patients in this study reported tumors in the lower extremities. More patients had previous surgery for TGCT (70.4% vs. 52.5%) and previous systemic therapy (25.9% vs. 8.3%) in our study compared with patients in ENLIVEN.^15^ Median time from TGCT diagnosis to pexidartinib initiation was slightly longer, and rates of surgery and off-label imatinib use were higher among the studied patients in the real-world setting compared with ENLIVEN. These findings suggest that the unmet medical need among some of the TGCT patients for effective systemic treatment might be fulfilled by the availability of pexidartinib.

About half of the patients initiated pexidartinib with a starting dose of 800 mg/day, while the other half reported lower doses at treatment initiation. This might reflect the comfort level of treating physicians: some might prefer using a full dose to achieve fast tumor response while others might emphasize tolerability at the beginning and the desire to avoid any risk of the potential for cholestatic hepatic toxicity. Dose reduction was reported in 44.6% of survey respondents (29 participants reported dose reduction from initial dosage and 8 reported dose reduction after titrating up from initial dosage), higher than the rate of dose reduction/interruption in ENLIVEN (38%).^15^ While 5 patients reported stopping pexidartinib because of symptom improvement, the most common reasons leading to treatment discontinuation or withholding included physician suggestion, abnormal laboratory results, and side effect. The common adverse events reported by these patients included alopecia, abnormal liver enzyme tests, and fatigue. It is critical to weigh the risks and benefits of pexidartinib and closely monitor patients on the drug in cases in which dose reduction or discontinuation becomes necessary.^15^ The findings also highlight the flexibility in dosing pexidartinib based on goals of care, clinical response, and patient tolerability.

Despite the early discontinuation or withholding of pexidartinib observed in some patients, the majority of patients reported improvement in overall symptoms and physical function with pexidartinib treatment. Reduction in Worst Stiffness NRS and Worst Pain NRS was similar to the reduction reported in ENLIVEN.^15,16^ According to the psychometric analysis of the Worst Stiffness NRS in TGCT patients and the correlation with tumor size,^24^ thresholds of ≥1 point for the Worst Stiffness NRS are considered clinically meaningful, which was achieved by 73.2% of the survey respondents after taking pexidartinib (Fig. 3). In addition, subgroup analysis was performed on patients stratified by duration of treatment of pexidartinib (<12 months and ≥12 months), the 2 subgroups shared similar trend of improvement in terms of patient-reported overall impression of change on symptoms and physical function (Supplementary Table S1), and reduction in worst stiffness NRS and worst pain NRS (Supplementary Table S2).

In this study, we researched on the real-world experience of pexidartinib based on data collected from 83 patients who completed the survey, and we compared their demographic characteristics with 171 patients who were invited but did not complete the survey, there was no statistically significant difference identified between the 2 groups (Supplementary Table S3). Thus, the results of survey respondents can be interpreted as representative of all pexidartinib patients invited to the survey.

A number of limitations should be considered when interpreting the results of this study. In general, cross-sectional studies lack the temporal link between outcome and exposure because both are examined at the same time and causal relationships might not be inferred. Participants were asked to recall past and present symptoms, which allows the evaluation of treatment effectiveness. Due to patients having to recall past symptoms, concomitant medication use, and physical therapy, this cross-sectional survey could be subject to recall bias. It is also difficult to control for confounding in cross-sectional studies. Survey data were collected from patients only; therefore, objective assessment of clinical outcomes, such as tumor response and disease progression, was not possible. Because the median time on pexidartinib was 6 months among respondents at the time of survey completion, long-term outcomes need to be evaluated in future studies. Finally, although there were observed reductions in use of prescription medications and physical therapy while taking pexidartinib compared with the time before treatment initiation, the reduction in resource utilization cannot be fully elucidated without detailed clinical and pharmacy data (eg, change in dosage and/or total number of analgesics).

## Conclusion

The similarity of demographic and disease characteristics between the survey respondents and patients in ENLIVEN suggests that patients receiving pexidartinib for the treatment of TGCTs in real-world practice were well represented in ENLIVEN. The longer time from diagnosis to pexidartinib initiation and higher rates of surgery and off-label imatinib use in the real-world setting compared with ENLIVEN suggest that there is an unmet medical need for effective systemic treatment fulfilled by the availability of pexidartinib. Similar to ENLIVEN, the majority of patients in this real-world study reported clinically meaningful improvement in overall symptoms and physical function during treatment with pexidartinib. Patient-reported symptom improvement was supported by reduced use of prescription medications and physical therapy. Findings from this cross-sectional survey confirm the benefit of pexidartinib in improving symptoms and functional outcomes among patients with symptomatic TGCTs from the patients’ perspective. Future research is warranted to examine the long-term benefits and risks of pexidartinib.

## Supplementary Material

Supplementary material is available at *The Oncologist* online.

oyad282_suppl_Supplementary_Table_S1

oyad282_suppl_Supplementary_Table_S2

oyad282_suppl_Supplementary_Table_S3

## Data Availability

The data underlying this article are available in the article.
