# Supplementary material for: Real-World Patient Experience of Pexidartinib for Tenosynovial Giant-Cell Tumor
Source: Oncologist. 2023 Oct 24;29(4):e535–43. doi: 10.1093/oncolo/oyad282 (PMC10994266; doi:10.1093/oncolo/oyad282)
Supplement: oyad282_suppl_Supplementary_Table_S1 [file oyad282_suppl_supplementary_table_s1.docx]

**Supplementary Table 1. Patient-Reported Overall Impression of Change on Symptoms and Physical Function, by Duration of Treatment of Pexidartinib***

|  | **Survey responders**  **with < 12 months of treatment with Pexidartinib**  **(N=56)** | **Survey responders**  **with ≥12 months of treatment with Pexidartinib**  **(N=24)** |
| --- | --- | --- |
| PGIC on overall symptoms since initiating pexidartinib, n (%) |  |  |
| Very much improved | 13 (23.2) | 10 (41.7) |
| Much improved | 16 (28.6) | 7 (29.2) |
| Minimally improved | 13 (23.2) | 4 (16.7) |
| No change | 10 (17.9) | 2 (8.3) |
| Minimally worse | 2 (3.6) | 0 (0.0) |
| Much worse | 1 (1.8) | 0 (0.0) |
| Very much worse | 1 (1.8) | 1 (4.2) |
| PGIC on PROMIS-PF since initiating pexidartinib, n (%) |  |  |
| Very much improved | 16 (28.6) | 9 (37.5) |
| Much improved | 13 (23.2) | 9 (37.5) |
| Minimally improved | 13 (23.2) | 3 (12.5) |
| No change | 11 (19.6) | 2 (8.3) |
| Minimally worse | 1 (1.8) | 0 (0.0) |
| Much worse | 1 (1.8) | 0 (0.0) |
| Very much worse | 1 (1.8) | 1 (4.2) |

PGIC, Patient Global Impression of Change; PROMIS-PF, Patient-Reported Outcomes Measurement Information System – Physical Function; SD, standard deviation.

* Among all 83 patients, only 80 patients included for the subgroup analysis (two patients missing duration of treatment of pexidartinib, and one patient did not report Worst Pain NRS or Worst Stiffness NRS).
