# Supplementary material for: Real-World Patient Experience of Pexidartinib for Tenosynovial Giant-Cell Tumor
Source: Oncologist. 2023 Oct 24;29(4):e535–43. doi: 10.1093/oncolo/oyad282 (PMC10994266; doi:10.1093/oncolo/oyad282)
Supplement: oyad282_suppl_Supplementary_Table_S2 [file oyad282_suppl_supplementary_table_s2.docx]

**Supplementary Table 2. Change in Worst Stiffness NRS and Worst Pain NRS before and after starting pexidartinib, by Duration of Treatment of Pexidartinib***

|  | **Change in Worst Pain NRS** | |  | **Change in Worst Stiffness NRS** | |
| --- | --- | --- | --- | --- | --- |
|  | **Survey responders**  **with < 12 months of treatment with Pexidartinib**  **(N=56)** | **Survey responders**  **with ≥12 months of treatment with Pexidartinib**  **(N=24)** |  | **Survey responders**  **with < 12 months of treatment with Pexidartinib**  **(N=56)** | **Survey responders**  **with ≥12 months of treatment with Pexidartinib**  **(N=24)** |
| **mean** | -2.71 | -3.50 |  | -3.11 | -3.29 |
| **SD** | 2.56 | 3.05 |  | 2.72 | 4.01 |
| **median** | -2 | -3.5 |  | -3 | -4 |
| **min** | -9 | -9 |  | -9 | -10 |
| **max** | 0 | 2 |  | 0 | 6 |
| **n** | 56 | 24 |  | 56 | 24 |
| **paired t-test p-value^#^** | <0.000001 | 0.000010 |  | <0.000001 | 0.000527 |

* Among all 83 patients, only 80 patients included for the subgroup analysis (two patients missing duration of treatment of pexidartinib, and one patient did not report Worst Pain NRS or Worst Stiffness NRS).

# Paired t-test comparing Worst Pain NRS and Worst Stiffness NRS before taking pexidartinib versus during pexidartinib treatment.
