# Supplementary material for: Real-World Patient Experience of Pexidartinib for Tenosynovial Giant-Cell Tumor
Source: Oncologist. 2023 Oct 24;29(4):e535–43. doi: 10.1093/oncolo/oyad282 (PMC10994266; doi:10.1093/oncolo/oyad282)
Supplement: oyad282_suppl_Supplementary_Table_S3 [file oyad282_suppl_supplementary_table_s3.docx]

**Supplementary Table 3. Demographic Characteristics of Survey Respondents and Non-respondents**

|  | **Respondents**  **(N=83)** | **Non-respondents (N=171)** | **P-value^b^** |
| --- | --- | --- | --- |
| Age^a^, mean (SD), year | 44.2 (14.1) | 46.4 (15.4) | 0.274 |
| Sex, n (%) |  |  | 0.269 |
| Female | 52 (62.7) | 100 (58.5) | - |
| Male | 30 (36.1) | 71 (41.5) | - |
| Missing | 1 (1.2) | 0 (0.0) | - |
| Race, n (%) |  |  | 0.561 |
| White | 54 (65.1) | 106 (62.0) | - |
| Black or African American | 7 (8.4) | 18 (10.5) | - |
| Asian | 6 (7.2) | 13 (7.6) | - |
| Native American | 0 (0.0) | 2 (1.2) | - |
| Native Hawaiian or other Pacific Islander | 0 (0.0) | 3 (1.8) | - |
| Other | 15 (18.1) | 29 (17.0) | - |
| Missing | 1 (1.2) | 0 (0.0) | - |
| Geographic location, n (%) |  |  | 0.080 |
| Northeast | 18 (21.7) | 50 (29.2) | - |
| Midwest | 22 (26.5) | 26 (15.2) | - |
| South | 20 (24.1) | 41 (24.0) | - |
| West | 21 (25.3) | 54 (31.6) | - |
| Other | 1 (1.2) | 0 (0.0) | - |
| Missing | 1 (1.2) | 0 (0.0) | - |

^a^Age was extracted from the Turalio REMS program patient enrollment forms.

^b^P-values are derived by two sample t-test for continuous variables or Chi-square test for categorical variables.

REMS, Risk Evaluation and Mitigation Strategy; SD, standard deviation.
